# The First Case of Burkholderia cepacia-Induced Myasthenic Crisis

**DOI:** 10.7759/cureus.45439

**Published:** 2023-09-18

**Authors:** Ali Şahin, Mehmet Zahit Aydın, İbrahim Furkan Asiltürk, Huseyn Babayev, Şerefnur Öztürk

**Affiliations:** 1 Department of Neurology, Selcuk University Faculty of Medicine, Konya, TUR; 2 Department of Neurodevelopers, Silicosome Biotechnology, Konya, TUR; 3 Swiss Institute of Allergy and Asthma Research (SIAF), University of Zurich, Davos, CHE

**Keywords:** bacterial pneumonia, neuroautoimmunity, burkholderia cepacia, myasthenic exacerbation, myasthenia gravis (mg)

## Abstract

We report the case of a 62-year-old male patient with a previous diagnosis of myasthenia gravis who experienced a myasthenic crisis due to a lower respiratory tract infection caused by *Burkholderia cepacia*. The patient was admitted to the neuro-intensive care unit and received ventilatory support to address respiratory insufficiency. Treatment included tigecycline and piperacillin-tazobactam for the suspected bacterial infection, as well as targeted management for the myasthenic crisis. Following a successful recovery and favorable clinical response, this case report aims to contribute to the literature by discussing the patient’s presentation and exploring the incidence and characteristics of *B. cepacia*-related myasthenic crisis.

## Introduction

Myasthenia gravis (MG) is a neuro-autoimmune disorder that impacts the neuromuscular junction. In MG, autoantibodies target antigens at the neuromuscular junction, leading to impaired neuromuscular transmission. While the exact cause of MG is not well understood, factors such as genetic predisposition, age, and gender may contribute to an individual’s risk of developing the condition. Patients with MG may develop autoantibodies against the acetylcholine receptor, muscle-specific kinase (MuSK), and low-density lipoprotein receptor-related protein 4 at the neuromuscular junction. However, some MG patients do not exhibit detectable autoantibodies, a condition known as seronegative MG. The prevalence of MG is approximately 150-250 patients per million, with an annual incidence of 8-10 cases per million [1].

Common MG symptoms include ptosis, diplopia, difficulty making facial expressions, problems chewing and swallowing, slurred speech, muscle weakness, and fatigue. Diagnosis typically involves clinical history and examination, followed by autoantibody detection and electromyography for confirmation [2].

MG is a chronic condition necessitating continuous management, yet with proper treatment and lifestyle adjustments, a substantial number of patients can achieve and maintain a satisfactory quality of life. However, MG can sometimes lead to complications such as myasthenic crisis, a potentially life-threatening condition that requires prompt medical intervention [2]. The primary goal of MG treatment is to compensate for acetylcholine-dependent signaling and regulate the immune system responsible for autoantibody production. By inhibiting acetylcholine esterase, the enzyme that degrades acetylcholine, pharmacological treatments (pyridostigmine and neostigmine) increase acetylcholine concentration in the synaptic cleft. In addition to pharmacological treatments and immunomodulatory agents, other treatment options such as plasmapheresis, intravenous immunoglobulin (IVIg), and thymectomy may be considered, depending on the severity of the condition and the patient’s response to initial treatments. Immunomodulatory and immunosuppressive agents (azathioprine, mycophenolate mofetil, cyclosporine, tacrolimus, methotrexate, cyclophosphamide, and rituximab) prevent the formation and function of autoantibodies due to immune system tolerance loss [3].

MG may develop if central tolerance loss occurs due to thymus-related issues. Thymectomy is another treatment option for MG. Approximately 10-30% of MG patients have thymoma [4]. The thymus plays a crucial role in MG as it provides central tolerance for T cells. Any thymus tissue pathology may disrupt T-cell biology and induce autoreactive T-cell formation. Thymic epithelial cells present self-antigens to T cells, which undergo negative selection (apoptosis) if they mount an immune response against self-antigens. Thymectomy can lead to symptom improvement or even remission in many MG patients, with varying degrees of success depending on factors such as age, disease duration, and the presence of a thymoma. Patients who undergo thymectomy at a younger age and with a shorter disease duration generally have better outcomes [5].

Myasthenic crisis, a severe complication of MG, can lead to life-threatening respiratory distress. Various factors can trigger a myasthenic crisis, including surgery, infections, pregnancy, therapeutic monoclonal antibodies, antibiotics, chemotherapy, and high temperatures. During a myasthenic crisis, oropharyngeal muscle weakness necessitates intubation and mechanical ventilation to protect the airway and maintain respiration [6].

Treatment for a myasthenic crisis involves providing respiratory support, immune regulation with immunomodulators/immunosuppressants, and addressing the underlying cause of the crisis. If an infection triggers the crisis, treatment may involve stopping or changing the drug (if drug-dependent) without using antibiotics which could worsen the myasthenic crisis. Certain types of antibiotics, particularly aminoglycosides, fluoroquinolones, and macrolides, have been reported to exacerbate MG symptoms and may trigger a myasthenic crisis [7].

*Burkholderia cepacia* is a type of bacteria known to cause respiratory infections, particularly in individuals with compromised immune systems or pre-existing lung conditions [8]. In this case report, we present an instance of a myasthenic crisis induced by *B. cepacia*, which has not been previously reported as a trigger for a myasthenic crisis. We aim to raise awareness among clinicians that *B. cepacia* could also be a causative agent in patients with a myasthenic crisis where an infectious origin is suspected.

## Case presentation

A 62-year-old male patient with a history of MG was taking 60 mg of oral pyridostigmine five times a day. He had no other remarkable medical or family history. The patient was first diagnosed with MG in 2015 and was prescribed a treatment regimen of pyridostigmine 60 mg (four times daily), methylprednisolone 64 mg (once daily), and azathioprine 50 mg (once daily). However, as the symptoms persisted, the daily dose of azathioprine was increased to 100 mg after two months. Within one year, the methylprednisolone dose was gradually reduced and eventually discontinued. Until 2023, the patient had regular pyridostigmine controls and did not experience any symptomatic events. In January 2023, he presented to the neurology clinic with fatigue and difficulty swallowing, requesting rituximab treatment. A serology examination revealed the presence of autoreactive antibodies against muscle-specific kinase.

**Figure 1:**
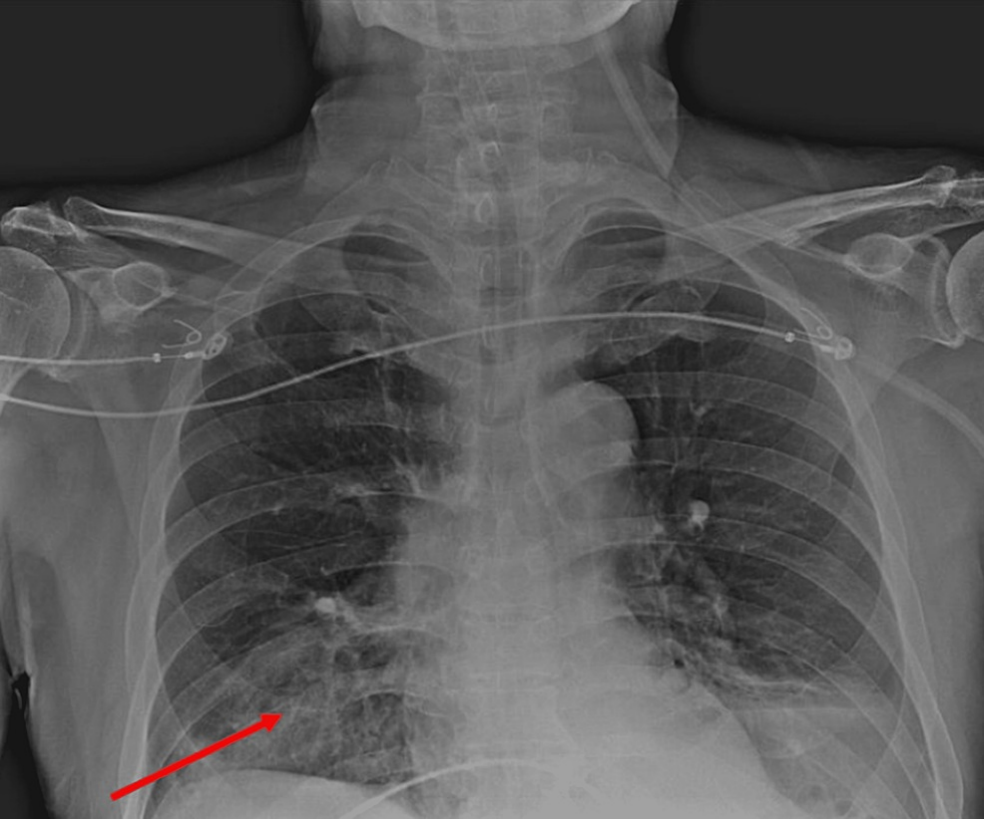
Chest X-ray of the patient. The patient has consolidations in the right lung on the chest X-ray.

**Figure 2:**
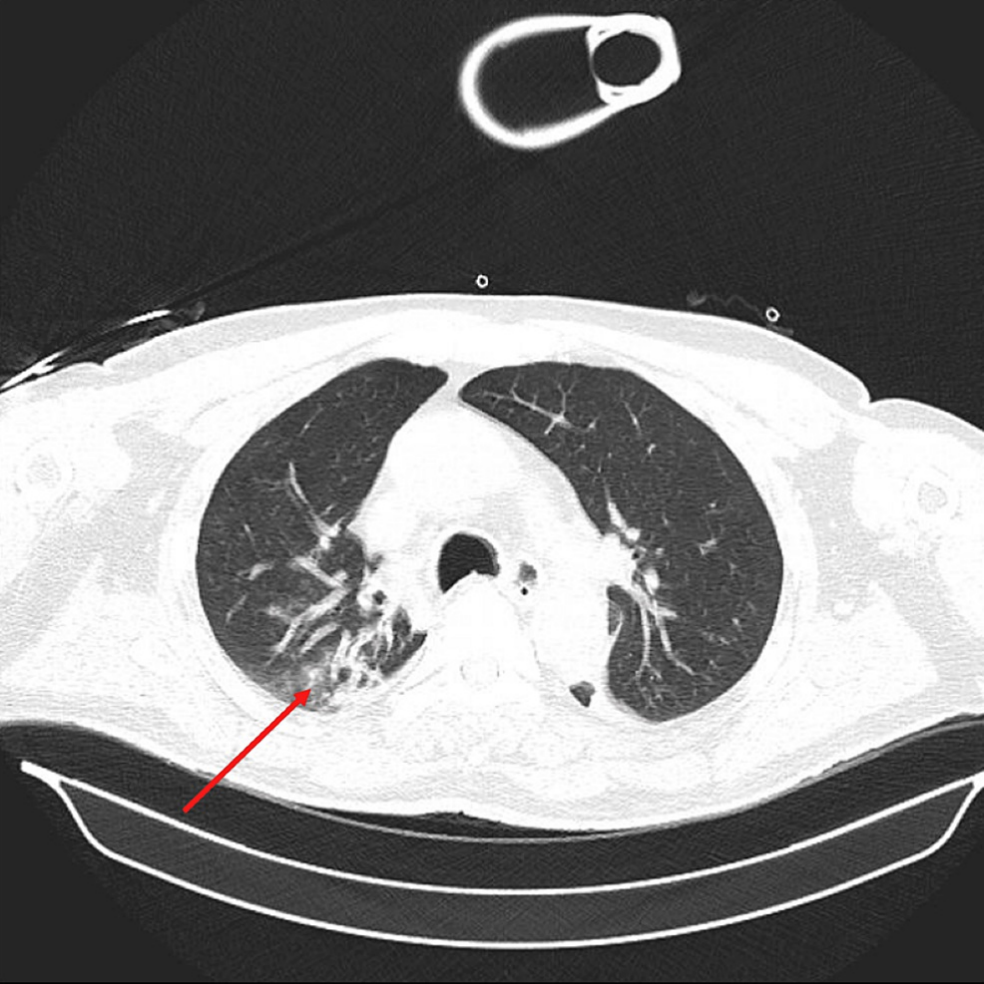
Pulmonary computed tomography of the patient.

In March 2023, the patient was admitted to the emergency medical clinic with dyspnea and general deterioration. Chest X-ray imaging showed consolidations in the right lower zone (Figure 1), and pulmonary computed tomography (CT) revealed a ground-glass opacity in the right lower zone (Figure 2).

No thymoma-related lesions were observed on radiologic examination. Initial blood investigation results indicated an ongoing myasthenic crisis (Table 1) with marked granulocytosis and elevated glucose levels. Lymphopenia, potentially due to the ongoing infection, and stress-induced hyperglycemia were also observed. No abnormalities in hepatic and renal functions were found, and electrolytes were within the normal range. Arterial and venous blood gas measurements (Table 2) revealed metabolic acidosis.

**Table 1:** Blood investigations of the patient.

Blood investigations	Results	Reference value
Neutrophil count	8.94 K/μL	1.7–7 K/μL
Lymphocyte count	0.55 K/μL	0.9–2.9 K/μL
Eosinophil count	0.00 K/μL	0.05–0.5 K/μL
NE%	90.9%	37–80%
LY%	5.6%	10–50%
Glucose	328 mg/dL	72–106 mg/dL
Alanine aminotransferase	78 U/L	0–41 U/L
Aspartate aminotransferase	48 U/L	0–32 U/L
Creatinine	0.69 mg/dL	0.7–1.2 mg/dL
Chlorine	93 mmol/L	98–107 mmol/L
Phosphorus	5.5 mg/dL	2.5–4.5 mg/dL
Aspirate culture and sensitivity	*Burkholderia cepacia* (trimethoprim-sulfamethoxazole and meropenem sensitive)	

**Table 2:** Arterial and venous blood gases results of the patient.

Blood investigations	Results	Reference value
Arterial pH	7.067	7.35–7.45
Arterial pCO_2_	148 mmHg	32–48
HCO_3_^-^	40.6 mmol/L	21.2–28.3
Venous pH	7.106	7.32–7.43
Venous pCO_2_	131 mmHg	38–55
Venous pO_2_	54.2 mmHg	17–53

The patient was transferred to the neurology intensive care unit due to worsening respiratory distress. He was intubated for respiratory support, and invasive ventilation was initiated. A bacterial culture of the patient’s respiratory aspirate showed the growth of *B. cepacia*. Antibiotic resistance studies demonstrated sensitivity to trimethoprim-sulfamethoxazole and meropenem and resistance to ceftazidime.

Following transfer to the neurology intensive care unit, the patient was treated with tigecycline 50 mg twice a day, piperacillin/tazobactam 4.5 g three times a day, pantoprazole 40 mg once a day, pyridostigmine 60 mg six times a day, prednisolone 5 mg four times a day, and enoxaparin 4,000 IU/0.4 mL once a day. However, as the patient’s symptoms did not improve, five doses of 0.35 mg IVIg and two sessions of plasmapheresis were administered.

During treatment, the patient’s procalcitonin levels remained within the reference range, while C-reactive protein levels peaked at 91.9 mg/L in the neurology intensive care unit before decreasing to 3.22 mg/L. On the 12th day of treatment, extubation was performed, and the patient was able to breathe independently with room oxygen.

## Discussion

Myasthenic crisis is a life-threatening complication of MG, characterized by respiratory failure due to weakness in the oropharyngeal and diaphragmatic muscles. Respiratory failure is the main cause of death in myasthenic crisis, with 10-20% of MG patients experiencing a crisis, typically within the first two years of disease onset [9].

Various factors can induce myasthenic crisis, including systemic infections, pregnancy, antibiotics, metabolic disorders, monoclonal antibodies, tapering of immune-modulating drugs, emotional stress, hot environments, the sudden elevation of body temperature, and adverse drug reactions. Bacterial pneumonia is the most common inducer, followed by viral and bacterial upper respiratory tract infections [6].

In this case, the patient’s myasthenic crisis developed due to a bacterial infection, leading to respiratory failure. Treatment included invasive mechanical ventilation, antibiotics, immunomodulators, and a plasma exchange regimen. *B. cepacia*, a gram-negative, aerobic bacterium that survives in aqueous environments, primarily causes respiratory tract infections. Although it is not typically associated with severe life-threatening infections, *B. cepacia* can cause infections in immunodeficient patients or those with underlying conditions [8].

This case represents the first reported instance of a myasthenic crisis induced by *B. cepacia*. The initial treatment regimen’s failure, including antibiotics and escalating pyridostigmine and prednisolone doses, highlights the challenges in managing myasthenic crises complicated by infection. The eventual success of IVIg and plasmapheresis demonstrates the importance of considering these treatments as potential options for patients with refractory MG or those experiencing a myasthenic crisis [10].

## Conclusions

This study demonstrates that *B. cepacia* can also induce myasthenic crisis in bacterial pneumonia, which is the most common inducer of myasthenic crisis. *B. cepacia* has not been previously reported in the literature as a cause of the myasthenic crisis. Our findings highlight the importance of considering *B. cepacia* as a potential causative agent in bacterial pneumonia associated with myasthenic crisis. Further research and case reports on this topic would be valuable to better understand the relationship between *B. cepacia* and myasthenic crisis and to potentially inform future treatment and management strategies for patients with MG.
